# Landscape review about the decision to euthanize a compromised pig

**DOI:** 10.1186/s40813-024-00378-6

**Published:** 2024-07-20

**Authors:** J. Stoffregen, T. Winkelmann, B. Schneider, K. Gerdes, M. Miller, J. Reinmold, C. Kleinsorgen, K. H. Toelle, L. Kreienbrock, E. grosse Beilage

**Affiliations:** 1 Institute for Biometry, Epidemiology and Information Processing (IBEI), University of Veterinary Medicine Hannover, Foundation, Buenteweg 2, 30559 Hannover, Germany; 2 Field Station for Epidemiology, University of Veterinary Medicine Hannover, Foundation, Buescheler Str. 9, 49456 Bakum Hannover, Germany; 3 Institute for Animal Hygiene, Animal Welfare and Farm Animal Behavior, University of Veterinary Medicine Hannover, Foundation, Bischofsholer Damm 15, 30173 Hannover, Germany; 4 Centre for E-Learning, Didactics and Educational Research (ZELDA), University of Veterinary Medicine Hannover, Foundation, Buenteweg 2, 30559 Hannover, Germany; 5 ISN-Projekt GmbH, Kirchplatz 2, 49401 Damme, Germany

## Abstract

Timely euthanasia of a compromised pig in farming practice has been identified as a critical topic in veterinary medicine. The questions ‘why and when are pigs euthanized’ and ‘what influences the decision making process’ need to be answered to improve the situation. In the past five years, work addressing these issues has been published in the literature, however, a synthesis of the findings is missing. With the help of a quantitative and qualitative analysis, this paper has generated a landscape review to outline major topics, the role of clinical signs and further influences on the decision to euthanize a pig. Due to the quantitative content analysis, 58 topics have been identified with the role of *welfare* as a justification and *training* for caretakers in making euthanasia decisions as the most frequently mentioned. The qualitative analysis of why and when a pig is euthanized generated a set of clinical signs for organ tracts, and a set of categories influencing the decision making process. The results outline the need to increase research on details specific to understanding how clinical signs evolve over time before euthanasia. In summary, the analysis provides an overview of work in the field and ideas on how to close knowledge gaps in the future. Moreover, the article contributes to harmonize efforts in the field and underlines the need for more research about the care of compromised and injured pigs.

## Introduction

The euthanasia of a pig within farming is a topic of major interest for veterinary medicine because the decision on when to euthanize is often a challenge. Previous publications have found that for far too many pigs, the decision to euthanize has been carried out too late, resulting in undue suffering and pain experienced by the pig [1, 2]. Due to this, efforts and discussions about procedural and legal requirements to avoid unnecessary pain and suffering for a pig have increased [3–5].

More specifically, understanding why pigs are euthanized and what influences the decision have become major research questions in the field [6–8]. However, approaches to answering these questions seem to be separated from each other. On the one hand, publications focus on caretakers and assess their attitudes, knowledge level, training and available guidelines for euthanasia [9, 10]. In this regard, certain euthanasia techniques have gained a lot of attention as well [8, 11, 12]. On the other hand, studies are published that elaborate on typical diseases, assessment schemes and mortality rates to enhance knowledge about clinical signs that justify or require euthanasia within a certain time frame [1, 13].

Only a few publications aim at bridging the topics (i.e. [14]), and there is a critical need to synthesize existing information. This article aims to fill this gap using a scoping literature review. Following a qualitative and quantitative content analysis of the latest publications, the state of knowledge about when and why pigs are euthanized, and what influences the decision-making process, is outlined. Moreover, the results provide an overview of frequently discussed as well as neglected topics and how they interrelate. Readers can use the article as a starting point to get into the field, find literature for further reading and to generate future designs of research on the decision making process to euthanize a pig.

### Method

The review is oriented on a scoping review guideline [15] with a qualitative analysis design [16]. Hence, a qualitative analysis and a quantitative analysis were performed separately. A synthesis was performed subsequently to validate and complement the findings of the qualitative review. The sections of the article reflect the dual approach with the first two sections relating to qualitative analysis, and the section on landscape analysis relating to quantitative analysis. Both approaches will be described in the following.

### Qualitative analysis

To generate answers for the two research questions, a grid for documentation was generated to inform the selection criteria of publications during the search. The grid entailed among others the perspective and influences on euthanasia, reasons for euthanasia, barriers to decision making, and proposed solutions.

The search was performed in the portal VetSearch of the University of Veterinary Medicine Hannover which searches through PubMed, AGRIS, CABI, Web of Science, SCIELO, SpringerLink, Wiley and ScienceDirect among others. Final terms for the search included for pigs (“pig*” OR “hog*” OR “porcine” OR “swine” OR “boar*” OR “sow*” OR “piglet*” OR “weaner*” OR “livestock”) and for the euthanasia (“euthanasia” OR “euthani*” OR “mercy killing” OR “killing” OR “endpoint*” OR “pain”). The terms were combined both in the title (search version 1 (v1)) or alternate in the title and the subject search field (v2, v3). To include studies on clinical signs, another search (v4) was conducted with terms for mortality (“cause*” OR “death*” OR “dead” OR “died” OR “mortality” OR “post-mortem”) and (“spontaneous*”) combined with pig-terms in the title. Papers from January 2010 to May 2023 were included if published in English or German language. A screening for the fit of topic in the papers was performed (screening I) and subsequently, those focusing not on euthanasia in particular (but mainly on lethal control, pain, damaging management procedures such as piglet castration and tail docking) were discarded (see Table 1).

**Table 1:** Metrics of the search and selection process of papers

Search process	Version 1	Version 2	Version 3	Version 4
Term combination	Title: pig terms AND Title: euthanasia terms	Subject: pig terms AND Title: euthanasia terms	Title: pig terms AND Subject: euthanasia terms	Title: pig terms AND Title: mortality terms Text: (“spontaneous*”)
Results	554	770	507	163
Screening (I)	333	629	259	89
Screening (II)	22	9	4	3

The qualitative analysis of papers started with selecting statements that described reasons or influences on the decision to euthanize a compromised pig. Subsequently, the statements were re-organized in tables to generate overall categories that were discussed and reviewed by the authors.

### Quantitative analysis

The landscape review was performed with the help of a quantitative analysis in order to assess whether findings from the qualitative analysis can be supported or complemented. In addition, it was elaborated, how topics among the selected papers interrelate. For this step, papers from the qualitative analysis were reviewed again and selected for the frequency analysis if they were not in German language, not guidelines, or not subject to cross-reference (like a dissertation comprising three papers) and it was possible to extract the raw text given the publication format (*n* = 15). The frequency analysis was performed in the statistical program R^®^ (version 4.3.0, [17]). The base code for the frequency analysis in R^®^ was created by using the integrated development environment RStudio (version 2023.6.1.524, [18]) and the R-packages tidyverse [19], openxlsx [20] and tm [21]. The initial code for the R^®^ analysis was generated using ChatGPT 4 (paid version, Open-AI, 29.11.2023). The initial code was controlled and optimized by one author. The basic R^®^ - code, which is free for re-use, is provided in the supplements (Additional file 1 R Code) and a full documentation of the coding process can be obtained on demand.

The frequency analysis in R^®^ allowed us to generate a list of the most frequently mentioned keywords among papers. Once the keyword list was generated in R^®^, the appearance of keywords in the publications was checked qualitatively to assure that one keyword stands for one particular topic, i.e. the keyword frequency is meaningful and used in a similar context among coded papers. In this regard, a recoding was conducted and a cut was made at a count of 7 (i.e. a keyword appeared at least seven times among all papers) unless research goals led to include further keywords.

For the keyword-approval, the program f4analyse^®^ was used (f4 analyse, version 3.4.5 from dr. dresing & pehl GmbH). It allowed to check, mark, count keywords and export the results for further analysis. In this step, we decided to mark at least one (and the best) keyword in a paper. Hence, the count from the recoding process in f4analyse is not representative for the importance but the number of papers elaborating on the same topic related to a keyword. The overall keywords and results of the quantitative and recoding analysis process are provided in the supplements (Additional file 2: Keywords of the frequency analysis).

Once the keyword recoding was finalized, the association of keywords was analysed by importing results in the statistical program SAS^®^ (version 9.4M7, SAS Institute Inc., Cary, North Carolina, USA); the SAS^®^ code can be obtained on demand. With the help of SAS^®^, it was checked how topics relate, i.e. how often topics mentioned in a paper corresponded to another. It needs to be considered (with regard to the number of analysed papers and approach), that the nature of the analysis is explorative. An association thus represents a joint appearance of keywords in the selected papers but neither a causal nor a statistically significant relationship. The full heat map of associations is provided in the supplements (Additional file 3: Heat map).

Given the wealth of results from the qualitative and quantitative analysis, readers need to consider that the synthesis of findings in this article provides an overview of answers to the research questions and that some included papers may provide a higher level of detail in some respects. Readers are invited to use this article to gain an overview of most salient points and implications for future work as well as references for further reading.

## Results

### Why and when to euthanize a pig

This section summarizes clinical signs of a pig that appear prior to euthanasia in relation to spontaneously occurring diseases or injuries of certain organ tracts. In case that a time frame to decide upon euthanasia is defined, readers should bear in mind that the decision is always case-by-case, i.e. the time frame needs to be tailored to the individual animal of concern. Moreover, the discussion of the validity of summarized clinical signs and time frames at the end of this article should be taken into account.

### Synthesis of findings

Pigs with **damaged integument** (i.e. compromised skin, tail, ear or flank biting injuries and decubital ulcer) are reported in most publications. Depending on the attributes of the affected skin and pig, observers consider treatment, close examination and culling as a first step rather than euthanasia [6]. When conditions worsen, i.e. wounds remain bloody, become infected or necrotic over time, a pig shows a compromised general condition, or multiple locations are affected, euthanasia is considered [3, 11, 22]. In case of tail injuries that extend to the base of the tail, immediate euthanasia should be considered [3]. Related symptoms that suggest a time-point for euthanasia if treatment fails are secondary lameness or palsy [3, 4]. Comparing results for sows, boars, and fatteners (hereafter “categories of pigs”), a tendency to treat and wait for recovery by caretakers rather than euthanize is reported though specific clinical signs for orientation are not defined [6]. For sows, decubital ulcer is outlined as one particular condition needing more attention, since this symptom was often noted among euthanized and found dead pigs [5].

Concerning the **respiratory tract**, clinical signs which are mentioned in relation to euthanasia comprise dyspnea, weight loss and longer sitting [3, 4, 13], as well as a combination of compromised general condition, dyspnea, long hair, fever or cyanosis [3]. In general, authors suggest granting a chance for recovery under treatment in case of general respiratory disease symptoms [4, 6, 23]. Depending on the state of the affected pig, a mean euthanasia score of a survey among caretakers suggested to treat and wait for a period of 24 h [6]. Comparing groups of pigs, more details about symptoms and diseases are outlined for mature compared to younger pigs [5, 24].

Concerning the **gastrointestinal tract**, hernias, diarrhoea, prolapses and inflammatory conditions are discussed in the papers. When seeing hernias, observers assess the need to euthanize with regard to a combination of the size of the hernia (distance to ground, diameter [6, 25]), effect of the hernia on movement and state of injury (skin perforated, ulcerated, draining, [3, 6, 22]). Concerning diarrhoea, observers orient on attributes of the stool (bloody or diffuse, with straining) and systemic consequences like dehydration to make a decision [22, 23]. When seeing rectal prolapses, the (suspected) time of tissue being protruded and wounded (damaged, necrotic), and signs of long-term suffering like weight-loss, are relevant for observers to make a decision about euthanasia [6, 23]. In case of a suspected rectal stricture, a combination of the clinical signs: a hunched back, impact on defecation, distended abdomen and signs of compromised general condition (weight loss, long hair) are assessed to decide about euthanasia [3].

Comparing categories of pigs, adult pigs and sows in particular tend to suffer more frequently from inflammatory processes like peritonitis, ulcer or enteritis. For inflammatory gastro-intestinal reactions, clinical signs, including decreased activity and inappetence, were reported by observers prior to making a decision about euthanasia [13]. For younger age categories, diseases associated with vomiting or diarrhoea were mentioned more often. In a survey about euthanasia scores for GIT diseases, observers tend to treat and wait for recovery beyond 48 h as well as to rather cull than to euthanize. For prolapses, a 24 h time frame is suggested in order to treat and wait for recovery. Moreover, slight differences in ratings between younger and adult pigs are outlined [6] while clinical signs to observe during this time frame were not further specified.

Diseases and injuries of the **urinary tract** (cystitis, nephritis) were outlined only for sows in one paper and no specific signs or symptoms are reported [5].

Concerning the **locomotor tract**, infectious and non-infectious diseases like (peri-)arthritis, arthrosis, myositis, osteomyelitis, and fractures among others were mentioned. Lameness is a central clinical sign which raises the attention of observers. Interestingly, the kind of lameness is often hardly described in detail, and grades like ‘severely’ are difficult to interpret or to compare. Instead, the clinical signs: using a minor set of legs for movement, carping (using carpal joints for weight bearing or movement), extent of inability to stand (with / without help), time spent lying, as well as size and number of affected joints, can be used to describe a time point close to euthanasia [3, 5, 13, 26, 27].

In a survey about euthanasia scores, caretakers suggested waiting 24 h for signs of improvement under treatment when a pig appears to be non-weight bearing on a limb [6]. Another combination of clinical signs described as worth for treatment focuses on secondary clinical signs such as fever, a hunched back, a shortened stance phase, and infected or swollen joints, as well as biting lesions (indicating a pig is subject to increased biting or refrains from fleeing from the biting of pen mates [4]).

Distinguished from the discussions of the symptom ‘lameness’ may be the clinical sign of hind-leg weakness. A typical combination raising concerns among observers is: bites, skin lesions, swollen joints and sitting. Yet, when the combination of signs is observed, only one study suggests waiting for recovery instead of immediate euthanasia [3].

Among the reviewed literature, a fractured bone seems to be distinguished from pigs with clinical signs of ‘lameness’ as well [14]. Fractured bones are often considered criteria for immediate euthanasia. Interestingly, most publications provide no specific details about what clinical signs are observed, except that one study uses different grades (compound, obviously, suspected, [22]). When comparing groups of pigs, the most detailed descriptions are provided for adult pigs. For post-weaning pigs, damaged digits may be in focus for euthanasia when they are (severely) wounded [22, 23].

Diseases and injuries of the **nervous system** are not specified with regard to decision making about euthanasia. Clinical signs mentioned in relation to euthanasia comprise convulsions, circling or incoordination, nystagmus or head tilt [6]. Another study suggests that pigs with rowing movements in lateral recumbency, should be examined for the skin colour (pink versus pale) and the status of feed and water intake (filling of the belly, sunken flanks) to make a decision about euthanasia [3].

Concerning the **reproductive tract**, vaginal prolapses are mentioned as reasons for the euthanasia of pigs in general [6]. For sows, infectious conditions like metritis, pyometra, mastitis-metritis and agalactiae (MMA), apart from dystocia, vaginal and uterine prolapses are outlined as well [5, 6]. For untreated and necrotic prolapses, the need for timely or immediate euthanasia is outlined [11, 25], though the specific timing is debated [14].

For the **cardiovascular system**, diseases were outlined for adult pigs only (anemia, hypovolaemic shock, cardiac failure, and heart / lung inflammatory diseases [14]). For cardiogenic conditions in general, clinical signs observed by a few caretakers prior to euthanasia are lameness, lying and inappetence [5]. Similarly, the clinical signs of sitting and weight loss were outlined as relevant in case of diagnosed inflammatory conditions [13].

Another category of findings did not belong to a certain organ tract but described a condition of **systemic sickness and weakness**. Clinical signs combined in this state are poor body condition scores or emaciation (BCS-1), the loss of appetite, non-ambulatory and unable to take up feed and water [23, 25]. One study suggests that additional clinical signs relevant for euthanasia are extended hair, hunched back and hypothermia [3]. Associated with being non-ambulatory, this study further suggests to elaborate on signs such as biting lesions and ulcer [3]. Comparing groups of pigs, the vitality score seems to describe conditions of systemic sickness and weakness for new-born piglets. A vitality score below 3 is defined as a grade requiring euthanasia [28]. It builds upon clinical signs of pale or cyanotic skin, unstable movement, being unable to stand and having a moderately filled or empty belly [28].

Last but not least, several nonspecific diseases, injuries or clinical signs are outlined among the reviewed papers (miscellaneous, unknown, [5, 13]).

### Discussion about why and when to euthanize a compromised pig

The synthesis of findings shows that in most cases few specific diseases or injuries require immediate euthanasia (except, i.e. fractured weight-bearing bones, or necrotic uterine prolapses). Hence, the decision to euthanize a pig rather stems from an observed combination of clinical signs which allows judging the severity, or which displays prolonged pain or suffering of a pig [3, 6]. Clinical signs making a difference for observers can be grouped into the following categories: **wound severity** (reddened, bloody, necrotic, damaged), **signs of generalization** (fever, cyanosis, multiple locations, swelling, hypothermia, prolonged hair), **altered postures** (sitting, hunched back), **impact on gait** (impairment using legs, swollen joints, non-ambulatory, inability to stand), as well as the **impact on food and water intake** (sunken flanks, inappetence, weight loss); an observation which corresponds with and refines previous studies [27].

The first points to discuss in view of the findings relate to the kind of clinical signs reported in the papers. In general, most of these clinical signs appear to be well-identifiable and hardly specific for an organ tract. Moreover, a higher number of observed signs as well as a combination of the clinical signs including *weight loss*, *sitting* and *lameness* raise the attention of observers prior to euthanasia. A point that needs to be carefully interpreted is that some clinical signs, such as *non-ambulatory* or *BCS-1*, seem to be self-standing in many publications, i.e. when being observed, no additional clinical signs are needed to decide *against* euthanasia (see a discussion in [29]). This impression is highly critical for making a timely euthanasia decision since, in most cases, irreversible weight loss of a pig is based on a loss of muscles and fat. The process is likely observable for days (or even weeks) before a state of emaciation like BCS-1 appears. Hence, the signs of non-ambulatory or emaciation are not an indication of acute events, but represent a chronic process and poor overall condition. Again, the authors of this article emphasize that a state of BCS-1 as well as clinical signs that represent chronic conditions indicate that timely euthanasia has been omitted.

Concerning the reporting of clinical signs, a lack of details can be seen for the urinary, reproductive and respiratory tracts, for the nervous system as well for certain age and sex categories. While not every organ tract may have its own specific clinical sign, (or from the other perspective - one particular clinical sign is usually related to a variation of reasons), the lack of detail appears for commonly reported symptoms like lameness as well. Due to this, a systematic assessment and comparison of why and when individual pigs are euthanized can hardly be made at the moment.

Related to the lack of reported clinical signs is a lacking discussion about implications for treatment and handling of a compromised pig. One example is that pain related or specific clinical signs are rarely reported [26]. This is stunning since in jurisdictional terms pain is considered a reason for immediate euthanasia in its own respect [30]. Some of the reported symptoms, like non-weight bearing on a limb or necrotic shoulder ulcer, are certainly painful [31, 32]. At the moment, however, findings are not discussed in this respect. Not at last, since healing under pain treatment suggests better recovery rates [33], the implication of clinical pain signs for treatment protocols should be discussed in future studies. Furthermore, findings suggest that the description of clinical signs should align with reknown classifications of diseases and symptoms. Not at last, studies should moreover include in the analysis if treatment was provided and how clinical signs have evolved over time.

The time frame during which clinical signs are observed prior to euthanasia is indeed another major point to discuss. Similar to the lack of details about clinical signs, there is a lack of information when clinical signs have been observed and how the signs evolve over time. One study [6] has conducted a survey to associate clinical signs with euthanasia scores on a five point scale (“Euthanize immediately (score 1), treat and euthanize on-farm within 12 hours if no improvement (score 2), treat and euthanize on-farm within 24 hours if no improvement (score 3), treat and euthanize on-farm within 48 hours if no improvement (score 4), and do not euthanize and re-evaluate if condition worsens or cull (score 5)” [6, p.46]). The author has emphasized the great variability of answers among respondents and suggested that more details are needed to describe the time frame for recovery in order to harmonize decision making paths. To increase and harmonize the report of details, the frequency of assessments may need to be raised to control the overall condition of the pig, the drinking and feed uptake and behaviour in comparison to group mates [2, 4, 6]. While the judgement about clinical signs and behaviour naturally builds upon experiences and knowledge about common disease trajectories, the decision to euthanize needs to be tailored to the individual pig of concern. In this regard, the assumption to set a time frame of 48 h to observe signs of recovery needs to be questioned (cf. [6, 34]). Given the lack of information about the longitudinal profile of clinical signs and their combination in diseased or injured pigs, any time frame would be arbitrarily defined. More research efforts are needed that are open to assess development over time (in terms of hours, days, weeks, probably months). Only in this case, common disease trajectories as well as decision paths when observing a compromised pig over time can be corroborated in the future.

Additional implications of the qualitative analysis concern the harmonization of reported clinical signs. Apart from a documentation of a time frame, clinical signs should be reported as a single entity in addition to the clinical diagnoses after death (cf. [5]). Moreover, both the relative and absolute occurrence of symptoms in relation to a disease or injury needs to be reported (i.e. [3]) to allow for systematic reviews in the future.

Last but not least, studies should outline whether clinical signs are reported by veterinarians [1, 3] or by caretakers [13]. While in some studies caretakers seem to observe (or report) a different set of clinical signs than veterinarians [5], another study showed no difference in how farmers and veterinarians judge the state of a pig when the same picture or set of descriptive details is provided [3]. Hence, future studies need to assess if caretakers orient upon different sets of clinical signs for euthanasia if they are not specified a priori, or if certain clinical signs are omitted during the report (i.e. not recognized or considered relevant for reporting, [5]). Similarly, it needs to be assessed if a difference in reporting exists between those who develop the protocols for euthanasia and those who actually perform euthanasia.

A final point to discuss is the evidence of (frequently) reported symptoms like non ambulatory, weak or the loss of weight. At the moment, the amount of (quantitative) data is too low for a systematic or even a statistical evaluation of the impact of specific factors. Discussions about the medical significance of these factors in relation to a certain time-point (i.e. immediate euthanasia) thus need to be seen with caution. Initial studies dedicated to analysis of the role of certain symptoms for timely euthanasia have already been published [3, 6]. Apart from the timing, the validation of signs for the decision process is also of concern, since it is not yet clear whether reported signs appear to observers in stables or whether observers provide answers that represent legal obligations [6].

The decision process and influences on deciding about the euthanasia of a compromised pig will be focused on in the following sections. Findings will outline how the decision to euthanize is influenced by more than clinical signs of the animal alone. Hence, to better answer the question of why and when a pig is euthanized, research studies are needed that combine knowledge about influencing factors with an assessment of clinical signs in a pig to be euthanized over time.

### What influences euthanasia?

To euthanize a pig is a case-by-case decision that is made based on experiences and knowledge about clinical signs typical for the trajectory of the respective disease, injury or condition. The individual pig of concern is in focus of the observer and the goal to avoid unnecessary pain or suffering should drive the decision to both wait and treat, to cull (i.e. kill for meat production) or to euthanize. However, since euthanasia of a pig happens within a work context and by a particular person, it is not surprising that the decision may be shaped by additional factors. Findings from the literature in this respect have been analysed and grouped into a generic model of influences on the farm (on farm), of individual persons (individuals), and surrounding external factors (Table 2). The synthesis of results will be provided in the first step and a discussion of findings subsequently.

**Table 2:** Categories and sub-categories of influences on the decision to euthanize a compromised pig

Level	On farm	Individual	External aspects
Category	Animal of concern	Gender	Public expectations
sub-categories	*assessment conditions* *attributes of clinical signs*	-	-
Category	Organisational aspects	Knowledge	Economy
*sub-categories*	*management* *work context* *culture*	*skills*, *abilities* *experience* *training*	-
Category		Mental constitution	
*sub-categories*		*justification* *attitudes*, *feelings*	

### Synthesis of findings

### On the farm

Factors that influence the decision to euthanize a pig on the farm embrace the categories **animal of concern** and **organisational aspects**. With regard to the first category, **animal of concern**, the decision to euthanize may be influenced by factors of the sub-category assessment conditions that come into play when identifying a compromised pig (Table 3). A great role seems to be the timing of examinations which can be influenced by business (day of the week and the season in general) as well as pig oriented perspectives (i.e. certain phases or groups of pigs). Another factor is the conduct of assessments. A proper examination requires space and light to (re-)identify a compromised pig within the pen mates. Last but not least, logistical challenges such as a lack of hospital pens may influence the decision-making process. Hence, if observers have no chance to separate and treat the animal, the identification may be perceived as meaningless [6, 7].^1^ In sum, assessment conditions need to allow an early identification and care for a compromised pig.

**Table 3:** Influences (on the decision to euthanize a compromised pig) associated with the sub-category “assessment conditions on farm”

Factor	Subfactor	Description	References
Timing of assessment	Pig – oriented timing	Incidents in the pen like feed time, also phases like pregnancy / farrowing of a pig	[7, 13]
		High risk age groups like piglets of elderly sows	[6, 7]
	Business – oriented timing	Assessments at weekends or during holidays	[7, 24]
		Assessments during seasonal business like harvesting	[6, 13]
		Assessments during seasonal phases, i.e. weather conditions like summer time	[13]
Proper conduct of assessment		Need to look closely to find compromised pigs, pick up the animal to inspect it closely	[2, 7, 27]
		Organise the follow up / the monitoring of compromised pigs, update examination procedures	[7]
Consequences of assessment		Logistical challenges like a lack of hospital pens	[6, 7]

Another set of influences on the decision process relates to the sub-category attributes of clinical signs (Table 4). In particular, the factor validity of clinical signs is a major topic as a lack of agreement amongst observers is often outlined as a negative factor. Related to the validity of signs is the value of signs for grading the overall condition of a pig. Hence, clinical signs should allow scoring a state (i.e. the severity of a state) or expressing the development of a state which evolves in a pig over time (i.e. Body Condition Score).

**Table 4:** Influences (on the decision to euthanize a compromised pig) associated with the sub-category “attributes of clinical signs”

Factor	Description	References
Validity of clinical signs	Lack of validity results in disagreement of evaluators about time-point	[6]
	Lack of validity results in disagreement about the case / meaning of clinical signs	[26, 29]
	Interest in pre-defining / providing instructions to conduct a solid examination / evaluation	[22]
Gradability of clinical signs	Body Condition Score used to grade the condition	[5, 28]
	Clinical signs gain an additional severity rank (i.e. “severe” sign) to judge upon a case; recommendations to look for extreme findings	[22, 29]
	Multiple pathologies raise more attention than single clinical signs	[5, 22]
	Attenuation and duration of signs are discussed to judge upon a case	[22]

In the category **organisational aspects**, influences related to the management of the farm can be described (Table 5). More precisely, the role of documentation (for following up on how a pig evolves) and available protocols (providing comprehensive work instructions) to organise human resources on farms are often outlined to influence euthanasia decision processes. Negative influence seems to stem from a lack of resources in terms of records, instructions and programs helping personnel to stem the situation on the farm.

**Table 5:** Influences (on the decision to euthanize a compromised pig) associated with the sub-category “management on farm”

Factor	Description	References
Documentation	Records about animals, provided treatment, hospital management	[4, 5, 7]
Protocols	(Available) work instructions / regulations how to handle hospital pens and organize the handling of pigs	[4, 7, 11, 35]
	Language of materials like work instructions and euthanasia regulations	[36]
Human resources	Available personnel on farm to conduct euthanasia	[6, 35]
	Capable (authorized) personnel on farm to conduct euthanasia	[6]
	Management on farm which cares for its personnel	[6, 36]

Concerning the work context (Table 6) the size of pig herds is discussed as a factor influencing both the handling of animals as well as euthanasia decision making. However, whether it is a larger or smaller herd size that is associated with timely identification and euthanasia is not yet clearly defined. Potentially related to the number of animals on the farm is the perceived workload and (perceived) lack of time during work. Logistical challenges influence the decision to euthanize a pig as well, such as available facilities on farms and space for conducting euthanasia. Concerning technical aspects, the maintenance of equipment was often discussed as a negative influence and some methods require their own precautions, like space of a CO_2_ box to be filled.^2^

**Table 6:** Influences (on the decision to euthanize a compromised pig) associated with the sub-category “work context on farm”

Factor	Description	References
Size of pig herd	The size of a farm (small, medium, high) in relation to decision making	[2, 7, 9, 24]
Workload	The workload given the environment on farm and the perceived time constraint of involved persons	[7]
Logistics	Logistics for handling carcasses, the location of euthanasia equipment and technical sufficiency of the spot for euthanasia	[2, 6, 11]
Technical aspects	Improperly maintained equipment, technical errors, and malfunctioning techniques	[6, 11, 35]
	Euthanasia needs to wait until the carbon dioxide box is filled or pigs can be moved at once to the spot for euthanasia	[6]
	Physical demands (force) to handle euthanasia techniques, technique to handle bolt guns	[7]

Another set of influences relate to the culture on the farm (Table 7). One cultural trait can be described as being accountable, both related to the person who performs euthanasia according to his own standards, as well as to standards established within the farm. Another trait of culture on the farm can be described as a duality between a focus on persons or on pigs. Oriented on persons, decision makers may decide about euthanasia with regard to the expected toughness among peers [34, p.2]. Oriented on pigs, persons may decide about euthanasia with regard to the goal to save all pigs. Related to the duality of pig vs. person seems to be the duality of individuals vs. routines. Hence, it may be key to either take care of routines or the mass of (healthy) animals first, or to take care of a compromised pig first. With respect to routines, operational blindness was described as an influencing factor as well.

**Table 7:** Influences (on the decision to euthanize a compromised pig) associated with the sub-category “culture of involved persons on farm”

Factor	Description	References
Accountability	Stance among peers on farm towards false practices and delayed euthanasia	[6, 34]
	Responsibility and sense of discipline to conduct immediate euthanasia	[6, 7, 35]
Orientation pig vs. person	Person oriented - expectation of toughness among peers	[34]
	Pig oriented - importance to save pigs for a person	[7]
Orientation pig vs. herd	Goal is to care for the herd health first, breed and feed first, inspect and treat herd before euthanize individual pigs	[6]
	Focus on routine work and not pigs, first spend time on office duties, then do extra work for individual pigs	[7]
Operational blindness	There is no need to adapt routines, it has always been like this	[6, 7]

Summarizing, the influences on farm show that the decision to euthanize a pig is shaped by many factors - beginning with the identification of a pig and the handling of a pig, to how it is generally communicated about pigs and euthanasia at the workplace. The categories seem to interrelate, like the lack of (capable) personnel vis-à-vis the need for a certain number of (skilled) personnel for euthanasia techniques [6, 7, 35]. Yet, the outlined factors may on their own influence decision making processes and the timing of euthanasia as outlined for the choice of the method.

### Individual

Influences related to the individual person who decides about euthanasia comprise the categories gender (role of sex), knowledge (skills, experience and training) and mental constitution (justification, attitudes) (see Table 2).

The first category, gender (Table 8), suggests that the gender of a person influences the decision to euthanize a compromised pig though contradictory results have been published [7, 9, 10, 24, 37]. It is unclear at the moment whether a difference exists and if this difference has a qualitative effect on the decision making process.

Factors of the category knowledge emphasize the (in most cases implicit) hypothesis that more knowledge helps to improve decision making about the euthanasia of a compromised pig (Table 8). Knowledge, abilities and skills considered valuable in this respect are the ability to identify animals needing euthanasia as well as to evaluate species-specific behaviour. Furthermore, skills to decide about the further course and the ability to perform euthanasia (and perform related methods) were mentioned among others.

**Table 8:** Influences (on the decision to euthanize a compromised pig) associated with the categories “gender and knowledge” of involved persons

Category	Description	References
Gender	Role of gender of a person	[7, 9, 10, 24, 37]
Knowledge and skills	General knowledge set of a person	[9]
	Skillset for identification of compromised pigs	[5–7, 9, 12, 13]
	Ability to differentiate normal from abnormal of a pig	[5, 7, 23]
	Skillset to evaluate (detect, monitor, grade) conditions of a pig	[6, 7, 9, 12]
	Skillset to diagnose the disease of a pig	[5, 7]
	Decision-making about further course of a pig	[9]
	Ability to perform euthanasia of a pig	[35]
	Skillset to evaluate chance of recovery of a pig	[6, 7, 11]
	Knowledge how to use euthanasia methods / techniques at work	[7, 11, 24]
	Lack of licences for euthanasia methods	[7]

Similar to the influence of gender, the role of experiences is often ambiguously discussed (Table 9). The repetition of euthanizing a pig, for example, may either facilitate or increase the stress of a caretaker over time. Similarly, both a lack of experience as well as having to euthanize pigs frequently may have a negative influence on deciding about euthanasia. Bad experiences are often discussed as a negative influence, especially when related to a particular method or equipment used during the process. Considerations about training outline a similar pattern (Table 9). It is assumed that training generates knowledge and improves the decision making process, but which kind of training is meant is yet to be defined (training about certain topics, conduct of euthanasia or mode of training). Moreover, the effect of training is ambiguous since both a lack of training as well as having been trained may have a negative influence. More precisely, results suggest that knowing how to perform euthanasia *correctly* may increase the discomfort with a particular method, or may increase the pressure to perform (i.e. fear of failure to perform).

**Table 9:** Influences (on the decision to euthanize a compromised pig) associated with the sub-categories “experience and training (knowledge)” of involved persons

Sub-category	Description	References
Experience	Repetition of euthanasia over time, familiarity with euthanasia scenarios i.e. the situation, euthanasia gets easier over time	[9, 10, 12, 24, 35]
	General lack or bad experience with euthanasia situations, i.e. impact of previous exposure to scenarios, resistance to euthanize pigs among new colleagues	[7, 35]
	Bad experiences with (improper use of) methods for euthanasia such as bolt guns	[7, 34, 35]
Training	Training may impact confidence of a person conducting euthanasia, may enhance the knowledge about requirements for conducting euthanasia	[24, 36]
	Lack of training for caretakers and for veterinarians on how and when to conduct euthanasia	[12, 24, 35]

Factors in the category of mental constitution comprise the role of personal justifications as well as attitudes about the topic and conduct of euthanasia (Table 2).

The topic justification seems to summarize approaches to de-personalize the decision making process (Table 10). Justification may be gained when seeing euthanasia of an individual pig as an act of humanity or necessity to secure animal welfare. Another set of arguments emphasize that health parameters define the decision, i.e. it is unavoidable to euthanize a pig since no other option (treatment, culling) is viable within jurisdictional frames.

**Table 10:** Influences (on the decision to euthanize a compromised pig) associated with the sub-category “justification” of the decision

Factor	Description	References
Reasons of humanity	It is better to euthanize the animal than to let it die alone, i.e. euthanasia is an act of humanity	[6, 24, 35]
Necessity to euthanize	There are good reasons to euthanize a pig, i.e. the decision is necessary, and people acknowledge the importance to stop suffering	[7, 36]
	The decision to euthanize serves pig welfare	[22–24, 28]
Last resort	Culling is no option, treatment is no option or is not feasible, recovery of the pig is unlikely so euthanasia is the last resort	[2, 11, 27]

Concerning attitudes and feelings (Table 11), one influencing factor on the decision process is the feeling of people in charge to have failed when deciding for euthanasia of a pig. The decision contravenes the will to keep the animal alive and thus may provoke mental conflict [6, 36]. Another factor is the willingness of a person to perform euthanasia or to take the responsibility to make the decision. The personality of individuals is an influencing factor as well, so feeling confident or detached, or personality traits like empathy may shape the decision process. Another set of influences relates to the perceived discomfort due to the conduct of euthanasia or due to attributes of particular euthanasia techniques. In addition to that, the topics mental stress and emotional strain related to the conduct of euthanasia have gained a lot of attention as influencing factors in recent publications.

**Table 11:** Influences (on the decision to euthanize a compromised pig) associated with the sub-categories ”attitudes and feelings” of involved persons

Factor	Description	References
Feeling to have failed	Feeling to have failed because the task is not to kill but to care for a pig; have the desire to keep pigs alive, have hope for healing of a pig	[6, 35]
Willingness to euthanize	Lack of willingness to perform euthanasia	[6, 12]
	Willingness to take on the responsibility to euthanize	[6, 12]
Personality of involved persons	Confidence and comfort with euthanasia of pigs	[7, 9]
	Feeling detached from the decision to euthanize or from pigs	[9]
	Feeling empathy for pigs, having a caring nature	[6, 9]
	In general, personality traits in relation to euthanasia	[7, 10]
Discomfort with the conduct to euthanize	Discomfort with act of killing, feeling that euthanasia is aversive	[6, 24, 35]
	Personal discomfort to euthanize a certain animal category	[36]
Discomfort with a certain method of euthanasia	Belief in benefit of a euthanasia method for the animal to be euthanized	[24]
	Impact of manual blunt force when applied, interest in methods other than blunt force for euthanasia	[11, 35]
	Risk for the involved persons when conducting euthanasia	[11]
	Impact of aesthetics connected with conducting euthanasia	[6, 11, 24]
Mental / emotional strain	Personal feeling that euthanasia is difficult, stressing, an emotional topic, and results in cognitive dissonance	[6, 34–36]

The decision to euthanize should always be tailored to the pig of concern. However, the individual who is making the decision will make a difference in how the decision is made and the situation is handled. This statement is inevitable but the synthesis of research in the field has outlined which factors have been considered so far and which factors may need more specific assessment in the future. Moreover, the synthesis outlines that long-term studies or follow-up designs are promising to explore how attitudes evolve and how experiences and training impact decision making over time. At the moment, not enough data is available to allow for a systematic analysis but future studies may use this data to generate new hypotheses for research.

### External aspects

Another set of influences relates to external factors. One category describes the role of public expectations that stem from consumers, the market or evaluators of decisions about euthanasia on the farm (Table 12). Decision makers have concerns to be condemned for their evaluation of a pig (or inaction) and that they have to face (public) penalties.

**Table 12:** Influences (on the decision to euthanize a compromised pig) associated with the category “public expectations”

Factor	Description	References
Public picture of pig farmers	Opinion on euthanasia / death rates, hospital pens in public	[7, 38]
	Activists activity and media defining the public picture of individual pig farmers	[7]
Public ideas about pig welfare	Concerns of consumers and markets about welfare of pigs on farm / during euthanasia	[5, 23, 24]
Conviction of pig farmers	Risk of farmers of being seen and condemned for inaction in case of false decisions about euthanasia	[6, 7, 11]

Apart from expectations from the public, economic considerations can shape the decision making process (Table 13). Factors mentioned in the analysis addressed the cost of euthanasia as well as the loss of profit when the animal is killed instead of culled.

**Table 13:** Influences (on the decision to euthanize a compromised pig) associated with the category “economy”

Factor	Description	References
Costs of euthanasia	Cost of the procedure, or techniques for conducting euthanasia	[24]
Loss of profit due to euthanasia	Uneconomic animals result in low return to profit but good animals result in a loss of profit	[2, 6, 22, 23]
Economic pressure on farm	Role of economic considerations shape considerations about euthanasia	[2, 6, 7]

It is important to know about external factors when talking with caretakers about the decision to euthanize a pig. Interest from the public should not lead to waiting for euthanasia of a pig if immediate action is required. However, future guidelines and training sessions may use the findings of this analysis to discuss how to handle expectations from the public or external institutions in case of suspected conflicts.

### Discussion of influences on the decision to euthanize

The decision to euthanize a pig has to be tailored to the animal of concern. Yet, the results show that there is a breadth of influences on the decision process and it needs to be discussed how they can be grouped and understood.

In general, many of the influences serve to support a rationale for euthanasia from a human’s point of view. More precisely, it seems that the best time point for euthanasia is sometimes rather derived from the perspective of the person who is making the decision, than considering from the perspective of a pig, what it would choose for itself. Arguments of this kind would reflect anthropocentrism, where the killing of an animal raises concerns because the aims or benefits of a person are challenged [39, 40]. An example from the analysis is the argument to avoid euthanizing a pig because it does not fit the slaughterhouse protocol [6] or because an individual person is uncomfortable with visual aesthetics of the procedure [11]. However, not all factors can be subsumed under this ethical perspective. Defining the time point for euthanasia on the basis of clinical signs, for example, reflect the perspective of biocentrism, where the decision to euthanize an animal is derived from the interest and needs of a pig as a moral entity [40]. It is, of course, not possible to simply ask a pig what it would choose for the further course of its life. However, considerations on how to validate clinical signs to best indicate suffering and whether the animal on its own has resigned [3], show the aim to improve understanding about a pig in a similar vein.

Summarizing, there is a breadth of influences on the decision to euthanize a pig apart from clinical signs and these factors need to be further disentangled. As briefly discussed, future studies could assess how influences cluster with regard to ethical perspectives like anthropocentrism and biocentrism or related models and myths [40–42]. A likely benefit of this approach is the chance to sharpen a shared understanding about the meaning of euthanasia both for the animal and individual making the decision [39, 43, 44]. Moreover, discussions about ethical concepts involving moral stress during euthanasia of companion animals [43, 44] can be used to further complement the current work on euthanasia of pigs, where the role of attitudes and mental health of individuals making the decision play a prominent role in publications at the moment [6, 35, 36].

Irrespective of the analytical lens, another finding to be discussed is that most influences describe a negative impact on defining the best time point for euthanasia of a pig (an exception is for example, the role of empathy or training). Future studies should ask for both, enabling factors and barriers to shape the decision making process (cf. [35]). Mentioning the process, another finding to discuss is that certain factors appear to influence certain time points during the decision making process to euthanize a pig. For example, being able to identify, or having enough light to identify compromised pigs in stables, are factors likely to initiate a timely euthanasia decision process. Other categories, like culture and expectations from the public, however, are difficult to allocate at the moment. Most publications in this review do not specify when factors play a role (i.e. at what time point during the decision making process). In order to enhance knowledge about influences in the future, studies should specify a generic process and ask involved interviewees to associate factors if possible. To harmonize studies in the field, this article has generally outlined the influence on the decision making process. Additionally, deciding between waiting and treating, culling, (immediate) euthanasia, or even end of life care, may be more promising outcome variables to improve knowledge of the situation [2, 4, 44].

At the moment, the evidence about the role of certain factors is low. Only a few publications elaborate on influences with the help of quantitative measures (i.e. [9]). Maybe related to this, the findings appear to be ambiguous when discussing the role of influences on the process of deciding about pigs. Furthermore, the factors tend to be related as outlined above. Future studies that elaborate on influences on euthanasia decisions will help to increase understanding by assessing the interdependence with the help of a quantitative factor analysis.

### Landscape analysis

### Report of results

The quantitative analysis served to generate an overview of published topics related to the euthanasia of pigs and to complement the qualitative analysis. Overall, 58 topics were identified and the two most frequently mentioned topics turned out to be *training* and *welfare* (count ≥ 200).

The first keyword *training* summarizes that training is seen as the (missing) link between repeatable decision paths to euthanize, the correctly performed act to euthanize, and the personal care for mental health following the act of euthanasia. This impression is supported by topics associated with the keyword training. The most frequent combination (of the topic training with other keywords) was in 67.67% of the papers the difficulty to identify pigs (*identify*), concerns to perform euthanasia (*performing)*, and to conduct humane euthanasia (*humane)*. The keyword *identify* stands for problems of how to identify compromised pigs with the help of early identification criteria. The keyword ***performing*** stands for being uncomfortable with the act of euthanasia; a feeling that shall be assessed (communicated, surveyed) and overcome (training, protocols) to improve decision making. The keyword ***humane*** summarizes the idea of a respectful ending of an animals’ life in terms of performing the act without harm and to avoid unnecessary pain or suffering.

The second most frequent topic in the papers is ***welfare***, which outlines concerns about the timing of euthanasia. On the one hand, timely and correct euthanasia is an inevitable part of securing welfare, and due to this reason, people decide to perform euthanasia, even when feeling uncomfortable. On the other hand, the fact that euthanasia is – at least in part – quantitatively reflected in the mortality rates of a farm, raises concerns because high mortality rates can hardly be sold as a quantifiable result of welfare to the public (cf. [5]). Thus, the chance of increasing mortality rates when euthanizing pigs initiates a dilemma, which may lead people to avoid or delay the decision making.

With regard to associations, the topic *welfare* appears to be associated in 60% of cases with the topics *identify* (see above) and *sick-and-injured*. The latter topic is a commonly used phrase to describe or justify why a pig is euthanized. Summarizing, the topic of *welfare* outlines a great interest of individuals in well accepted reasons of why pigs are euthanized. It also brings in a slightly different focus than the topic training, despite the fact that the topics (welfare and training) are associated eventually. They illustrate the core conflict of a responsible person to handle the situation of euthanizing a pig correctly, reproducibly and accountable given personal influences and factors on the farm or from the public.

**Fig. 1:**
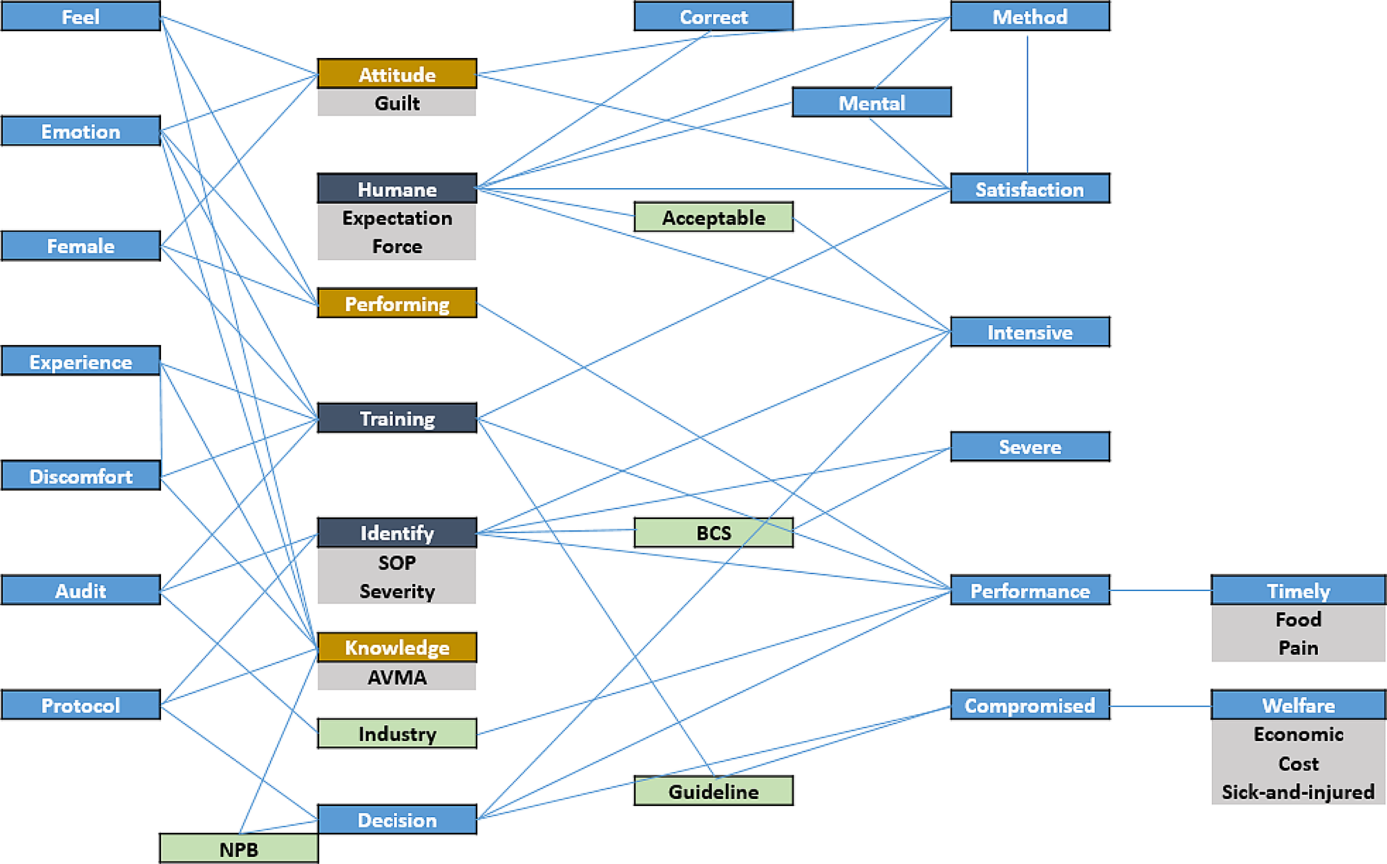
List of the keywords and their mutual appearance in the selected papers. The dark blue fields (humane, identify, training) are associated with nine other keywords. The yellow keywords (knowledge, performing and attitude) are associated with six other keywords. The light blue keywords are associated with several keywords, the green keywords are associated two and the grey keywords with one other keyword. Blue lines associate keywords that appear mutually in all of those papers that have mentioned this particular keyword. Abbreviations: SOP - standard operating procedure, AVMA - American Veterinarian Medical Association, NPB - National Pork Board, BCS - Body Condition Score.

With the goal of the article to provide an overview of the topics in the selected papers, the remaining set of keywords and associations should be taken into account (Fig. 1).

Figure 1 outlines most of the keywords of the quantitative analysis. The blue lines associate keywords that appeared mutually in those papers that have mentioned this particular keyword (mutual appearance; i.e. six papers that discussed the topic attitude have discussed the keywords guilt, feel, female, emotion, and so forth, as well). As a result, the analysis generates a set of clusters that appear to be mutually discussed in the selected papers.

An example of a cluster can be seen with regard to the keyword *compromised*, which is associated with the keywords decision making (*decision*), *welfare* and *guideline*. The association suggests a thematic cluster about the question of how to justify the decision to euthanize a pig. Another example can be shown for the keyword *industry*, which is associated with the keywords *audit* and *performance*. The association suggests a thematic cluster, which addresses economic interests in evaluating the euthanasia process on the farm from the perspective of industrial organisations. A third example is the association of the keyword timely euthanasia (*timely*) with the topic painless euthanasia (*pain*), concerns about culling pigs (*food*) and concerns to perform euthanasia *(performance)*. The association suggests that concerns about missing the time point to cull a pig, and to improve economic performance, are mutually discussed when addressing the topic of timely euthanasia in the selected papers.

Since Fig. 1 outlines the mutual appearances of keywords, readers need to consider that a topic like timely euthanasia (*timely*) is of course discussed with regard to the topics decision making (*decision*), *training* and *knowledge* as well. Figure 1 serves to outline dense associations in the selected papers, i.e. the topics that are mutually addressed in a paper when being mentioned. Yet, another combination of topics may have appeared as well. To get a full grasp of topics and associations, a heat map of associations has been generated (Additional file 3: Heat map).

Apart from focusing on dense associations, however, a landscape review requires assessing neglected keywords or those being weakly associated. In this respect, the analysis shows that only three of the selected papers elaborate on the keywords *hospital*, *compassion*, *fatigue*, *records*, and *risk*.

The topic *hospital* describes facilities used to monitor sick or injured pigs or the handling of a pig to reduce the risk of herd infections. The topic is discussed with regard to the topic of decisions to euthanize, keeping records about pigs (*records*) and the topic *risk*. The keyword *records* outlines that recording data on treatment of sick or injured pigs as well as causes of deaths are lacking despite that they are seen as the core basis for doing more research on euthanasia of pigs. This keyword supports previous suggestions in this article about generating clear documentation of the clinical signs and treatments of a pig before euthanasia. The topic *risk* evaluates the decision of separating or euthanizing a pig with regard to the chance of harming the pen mates. In sum, the association suggests a thematic cluster about the role of documenting a disease trajectory over time to evaluate how to proceed in case of recovery of a pig. Further details about the full list of keywords can be seen in the supplementary files (see Additional file 2: Keywords of the landscape analysis).

### Discussion of the findings of the quantitative analysis

In previous publications, the choice of topics for euthanasia training and assessment was discussed (cf. [12]). With regard to the least and the most frequently mentioned topics and associations in this study, results suggest that knowledge of how to identify compromised pigs, and how to evaluate harm during euthanasia may be fruitful. Moreover, the question of how to proceed in case a pig recovers raises interest, i.e. when and whether to join the pig with previous pen mates. With regard to the concerns about welfare, training may also address the questions of how to deal with dilemmas and moral conflicts. At the moment, only few publications elaborate on ethics or the role of mental health [6, 35] but the concerns should be further specified and even included in guidelines about euthanasia of a compromised pig. Last but not least, the thematic clusters of the quantitative analysis suggest that a suitable variable for training outcomes may be the effect on the confidence of trainees in deciding about the time point of euthanasia and confidence in performing euthanasia.

While interpreting the results of the quantitative analysis, readers have to bear in mind that the choice of integrated papers is selective, so results may not be representative of the whole body of published literature on the topic euthanasia of pigs. The landscape shows the most frequently mentioned and associated topics as well as those that are rather neglected in selected papers. Researchers who are new to the field can use the landscape to get into the field and to be sensitized about the multitude of topics and influences on decision making and how they may be interdependent.

To elaborate on the generalizability of results, further automated quantitative content analysis is needed in order to represent the landscape with greater breadth. Given that an automated content analysis requires specifying thematic terms and field specific stop – words, results of this study provide a starting point to compare gathered results in the future.

### Discussion and implications

The aim of the article was to elaborate on the questions of why and when pigs are euthanized and what influences the decision making about euthanasia. The data to answer the questions stem from an extensive literature review and a subsequent qualitative and quantitative data analysis. The purposeful selection of papers during the analysis requires caution because the results of this paper may not be fully representative of the whole body of literature about euthanasia of pigs. However, the goal was to generate a synthesis that helps researchers to get into the field, to refine assumptions about training or influences on decision making processes as well as to outline research gaps for future studies.

While being cautious of the generalizability, the synthesis has generated a list of clinical signs for different pig categories and organ tracts. Moreover, the combination of typical signs was discussed. The results emphasize that more research is needed to elaborate on details of clinical signs from the perspective of veterinarians and farmers, for certain sex and age categories (weaners, fattening pigs and boars), and organ tracts. While not every disease or disorder may have its own particular clinical sign for timely euthanasia, a list of clinical signs typical for the trajectory of respective diseases, injuries and conditions is important. It helps to train new caretakers in identifying pigs but also to outline assumptions about justifiable conditions of a pig over time. To improve the list of clinical signs generated in this analysis, future studies should elaborate on how clinical signs evolve over time to generate longitudinal profiles of typical disease trajectories.

When comparing results of the qualitative and quantitative analyses, the interest in research about clinical signs can even be more specified. Clinical signs should be evaluated for being valid as early identification indicators and for allowing objective and reliable assessments (easy to assess, facilitate inter-coder-reliable grading of the development of the condition of a pig). In this respect, findings also suggest to avoid grading a condition of a pig with descriptors like severe, higher, or more or less, in order to improve comparability of case reports in the future.

Concerning the second question, what influences euthanasia decision making, a set of categories and factors has been synthesized. The results show that a multitude of factors related to the individual pig, the person who decides about or performs euthanasia, and the situative context influence the decision making about euthanasia. The influences that are related to individual persons such as the sub-categories justification, attitudes, and mental health, have gained a lot of attention in the selected papers. Yet, economic concerns as well as influences related to the public picture emphasize that the topic timely euthanasia requires broader societal debate as part of pig welfare in stables. Influences on the decision making of euthanasia spread beyond the boundaries of veterinary medicine research. Hence, our knowledge and understanding of the topic will benefit from multi-disciplinary studies and perspectives in the future. In the field of ethics, for example, latest publications on euthanasia elaborate on ethical-boundary work, i.e. narratives how veterinarians handle and justify different cases of euthanasia [45]. Another approach may be to integrate social science perspectives to assess how consultancy formats between veterinarians and caretakers may benefit from assessing personality traits [46]. Summarizing the overall discussion, key points to advance knowledge about timely euthanasia of a compromised pig are:

- Conduct multi-disciplinary research to advance understanding about influences on timely euthanasia of a pig that go beyond the boundaries of veterinary medicine research; results of this article suggest to focus on concepts that disentangle the topics: ethical dilemma, personality traits, mental health, and validity of clinical signs.
- When researching on clinical signs that are typical for common diseases and injuries of compromised pigs, invite observers (farmers and veterinarians) to report in detail (the kind, time point, development of clinical signs in addition to the diagnosis and provided treatment), and to align the reporting with reknown disease / disorder classifications to harmonize the results, and to include diseases and injuries where only a few publications are available.
- Elaborate on training approaches or guidelines that raise confidence of caretakers and veterinarians in early identification and evaluation of compromised pigs and that sensitize about the multitude of influencing factors.

## Conclusion

Timely euthanasia of a compromised pig remains a salient topic which needs more attention from researchers, veterinarians and farmers in the future. A lot of work has been done to elaborate on influences on timely euthanasia and how the knowledge and skills about this topic can be improved. The landscape generated in this article is based on a synthesis of findings with the help of a qualitative and quantitative analysis. The results help to provide an overview of clinical signs reported with regard to typical reasons of euthanasia. In addition, a set of influences on the decision process and an overview of least and frequently mentioned topics, including their association in selected papers, are presented and discussed. Findings of this article allow delineating research gaps for future studies on the topic euthanasia of a compromised pig, but also serve as a starting point for researchers to get into the field.

## Data Availability

No datasets were generated or analysed during the current study.

## Footnotes

1 In Germany, a hospital pen must be available for sick or injured pigs. From the perspective of the authors, difficulties related to the handling of compromised pigs are not enough reason to delay the decision to treat and wait, to cull or to euthanize a pig.

2 The method for euthanasia should be appropriate to the animal of concern which embraces both the kind of method and time-point of conduct. For the authors of this review, waiting for a CO_2_-box to be filled is no reason to delay euthanasia of a pig.
